# Nerve Stimulation by Triboelectric Nanogenerator Based on Nanofibrous Membrane for Spinal Cord Injury

**DOI:** 10.3389/fchem.2022.941065

**Published:** 2022-07-15

**Authors:** Chaoling Xu, Fan Zeng, Danyu Wu, Pang Wang, Xiaoling Yin, Bin Jia

**Affiliations:** ^1^ Department of Neurosurgery, Chongqing General Hospital, Chongqing, China; ^2^ Chongqing Key Laboratory of Nonlinear Circuits and Intelligent Information Processing, College of Electronic and Information Engineering, Southwest University, Chongqing, China; ^3^ Shiyoulu Primary School, Chongqing, China

**Keywords:** spinal cord injury, nerve stimulation, triboelectric nanogenerator, nanofibrous membrane, TENG

## Abstract

Spinal cord injury (SCI) is a devastating and common neurological disorder that is difficult to treat. The pain can sustain for many years, making the sufferer extremely painful. Nerve stimulation was first reported half a century ago as a treatment for neuropathic pain. Since then, the method of electrical stimulation through leads placed in the epidural space on the dorsal side of the spinal cord has become a valuable therapeutic tool for SCI. But nerve stimulation equipment is expensive, and the stimulator design and treatment plan are complicated, which hinders its development. In recent years, wearable and implantable triboelectric nanogenerators (TENGs) developed rapidly, and their low cost and safety have brought a new turning point for the development of nerve stimulation. Nanofibrous membrane has been proved that it is a flexible material with the advantages of ultrathin diameter, good connectivity, easy scale-up, tunable wettability, fine flexibility, tunable porosity, controllable composition and so on. In this paper, we discuss the technology of using nanofiber membrane on clothing to create TENGs to provide continuous electrical energy for nerve stimulation to treat SCI in patients by analyzing previous research.

## Introduction

Spinal cord injury (SCI) is a devastating and common neurological disorder that has profound implications for modern society from a physical, psychosocial, and socioeconomic perspective (Dumont et al., 2001). The early stage of SCI is mainly treated by surgery, such as fracture reduction and internal fixation. Drug therapy can also be used: postoperative glucocorticoid pulse therapy to promote nerve repair. In addition, there are psychotherapy, rehabilitation and traditional Chinese medicine treatment (Kwon et al., 2004). In the nineties, SCI meant being confined to a wheelchair and lifelong comorbidities (McDonald and Sadowsky, 2002). Chronic pain is a significant problem of SCI and a major barrier to effective recovery (Siddall and Loeser, 2001). Electrical stimulation is a rehabilitation and therapeutic strategy that can help patients relieve or even heal pain, thereby treating spinal cord injuries. Nerve stimulation as a treatment for pain was first reported half a century ago. Since then, the use of electrical stimulation through wires placed in the dorsal epidural space of the spinal cord has become a valuable therapeutic tool for neuropathic pain and SCI (Shealy et al., 1967).

Nerve stimulation is the application of electrodes, which are directly or indirectly placed in the innervated area of ​​nerve tissue, or around the nerve root or the dorsal horn of the spinal cord, to stimulate the nerve. The device that emits nerve stimulation is called a neurostimulator. Its purpose is to improve the patient’s pathological state, clinical symptoms, and even achieve the effect of curing. And the current required for neurospinal electrical stimulation only needs to reach the microamp level (Ren et al., 2016; Fan and Sdrulla, 2020). Electrical spinal cord nerve stimulation therapy is a method of treating neuropathic pain and SCI by implanting electrodes into the spinal canal and stimulating spinal nerves with pulsed current. The pacemaker system sends weak electrical pulses to the spinal cord, blocking the transmission of pain signals from the spinal cord to the brain, effectively relieving intractable neuropathic pain, restoring physical function, and improving patients’ quality of life. Although electrical spinal cord nerve stimulation has been demonstrated significant benefits in the treatment of some neuropathic pain, its equipment is expensive, and the stimulator design and treatment plan are complicated, which has hampered the development of the technology (Jensen and Brownstone, 2019).

Academician Wang proposed the triboelectric nanogenerator (TENG) research concept in 2012. TENG convert mechanical energy from the environment into electrical energy, which can be used as sources of power or as a sensor signal (Cheng et al., 2019). The advantages of TENG include high efficiency, portability, cost-effectiveness, flexibility, wide range of material availability, and device miniaturization and so on (Feng et al., 2018). Since TENG was proposed, it has developed rapidly and aroused extensive research interests in the world. TENG has four basic working modes: vertical contact-separation (CS) mode, lateral sliding (LS) mode, single-electrode (SE) mode, and freestanding triboelectric-layer (FT) mode (Luo and Wang, 2020). Initially, researchers designed high-performance TENG by changing different materials. Then wearable triboelectric nanogenerator (WTENG) gradually became one of the new research hotspots in this field. Nanofiber is a wire-like material with a diameter of nanometer scale and a large length with a certain aspect ratio. In addition, the fibers that are modified by filling nanoparticles into ordinary fibers are also called nanofibers. Nanofibers have a wide range of uses. For example, implanting nanofibers on the surface of fabrics can form a stable gas film to make an amphiphobic interfacial fabric, which is waterproof, oil-proof and anti-fouling. The preparation in WTENG has great advantages. In 2015, Seung et al. designed a flexible, foldable nanopatterned WTENG with high power generation performance and mechanical robustness (Seung et al., 2015). WTENG can harvest human mechanical energy to power wearable electronics. In 2019, Wu et al. proposed a self-powered wearable iontophoresis system based on WTENG for relieving pain from ankle injuries (Wu et al., 2020). The invention of the TENG is a landmark discovery in the field of mechanical energy generation and automatic systems. This efficient collection of mechanical energy provides a whole new model (Tang et al., 2015a). The TENG energy system has laid a solid theoretical and technical foundation for the realization of integrated nanodevices and large-scale energy supply, and will be applied in many fields such as the Internet of Things, medical science, national defense and security, and may affect all aspects of human life. And the development and characteristics of TENG also have shown great potential in the medical field and electrical spinal cord nerve stimulation.

**FIGURE 1:**
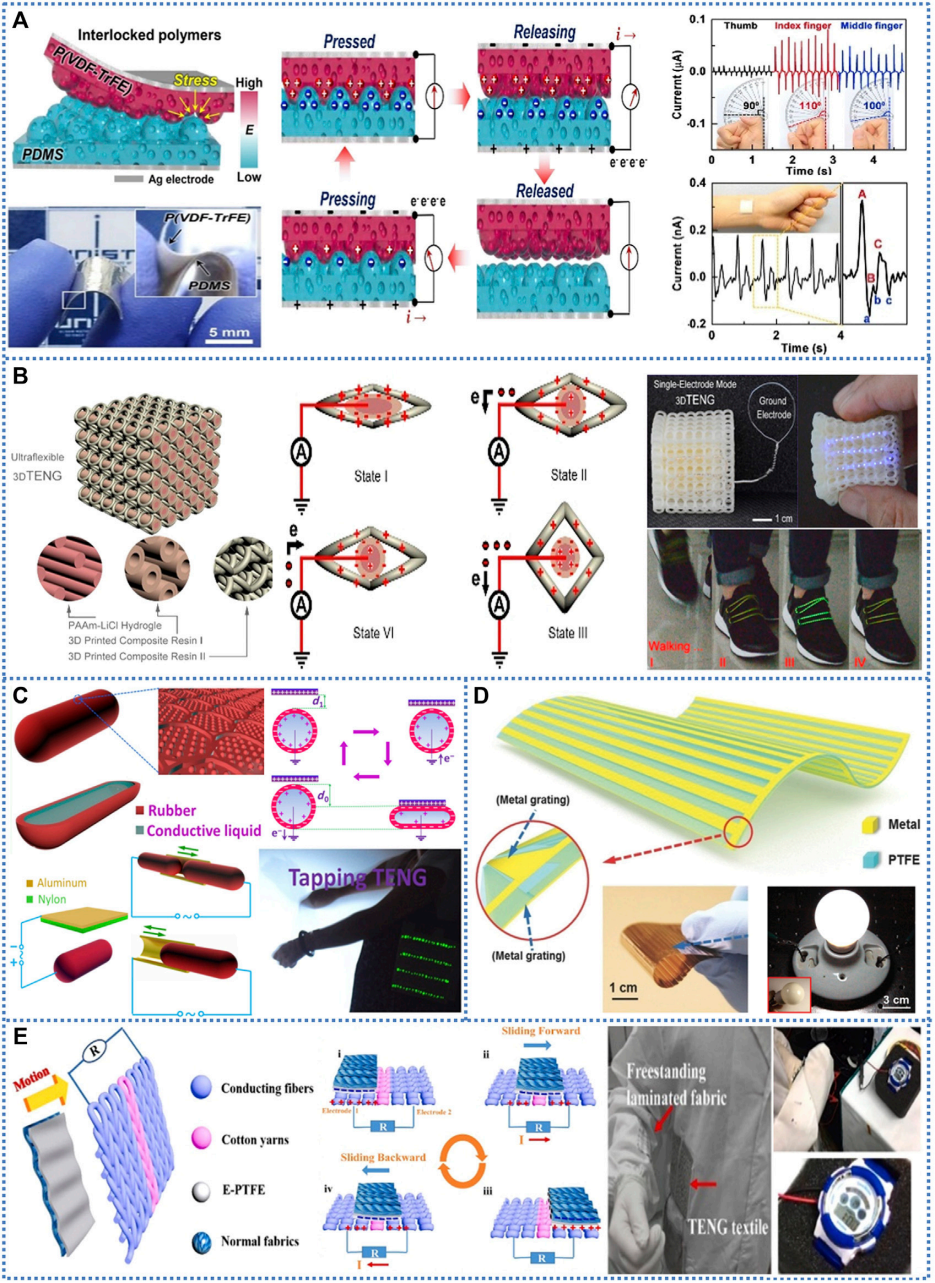
Various structures of soft TENGs. **(A)** Schematic of vertical contact-separation mode soft TENG with the interlocked micro-ridge structure. **(B)** The configuration of a single-electrode mode soft TENG with three-dimensional structure. **(C)** The single-electrode mode soft TENG with package structure. **(D)** The micro-grating based soft TENG in lateral sliding mode. **(E)** The wearable textile-based soft TENG in freestanding triboelectric-layer mode (Song et al., 2021).

In this paper, based on previous research of TENG in the medical field and nerve stimulation, we discussed the technology of using nanofiber membranes to create WTENG to harvest human mechanical energy to provide electrical energy for electrical spinal cord nerve stimulation on patients to treat SCI by alleviating and treating chronic pain. We consider using implantable electrodes to be placed directly around the spinal cord nerves, while establishing contact-separation TENG (which can be placed on elbows, knees) to collect the mechanical energy generated by human movement and convert it into electrical energy to stimulate the spinal cord nerves through electrodes (Figure 1 shows various WTENG models).

## Nanofibrous Membrane Constructed TENG for Electrical Spinal Cord Nerve Stimulation

### The Working Principle of TENG

TENG uses the surface charge generated by the contact of two dissimilar materials and time-varying electric field to drive the flow of electrons in an external circuit. When two dissimilar materials are in contact, their surfaces will generate positive and negative electrostatic charges due to the contact electrification. And when two dissimilar materials are separated due to the action of mechanical force, the positive and negative charges generated by the contact electrification will also be separated. The charge separation will correspondingly generate an induced potential difference between the upper and lower electrodes of the material. If a load or wire are connected between the two electrodes, this induced potential difference will drive electrons to flow between the two electrodes through an external circuit (Wang et al., 2015).

### WTENG

**FIGURE 2:**
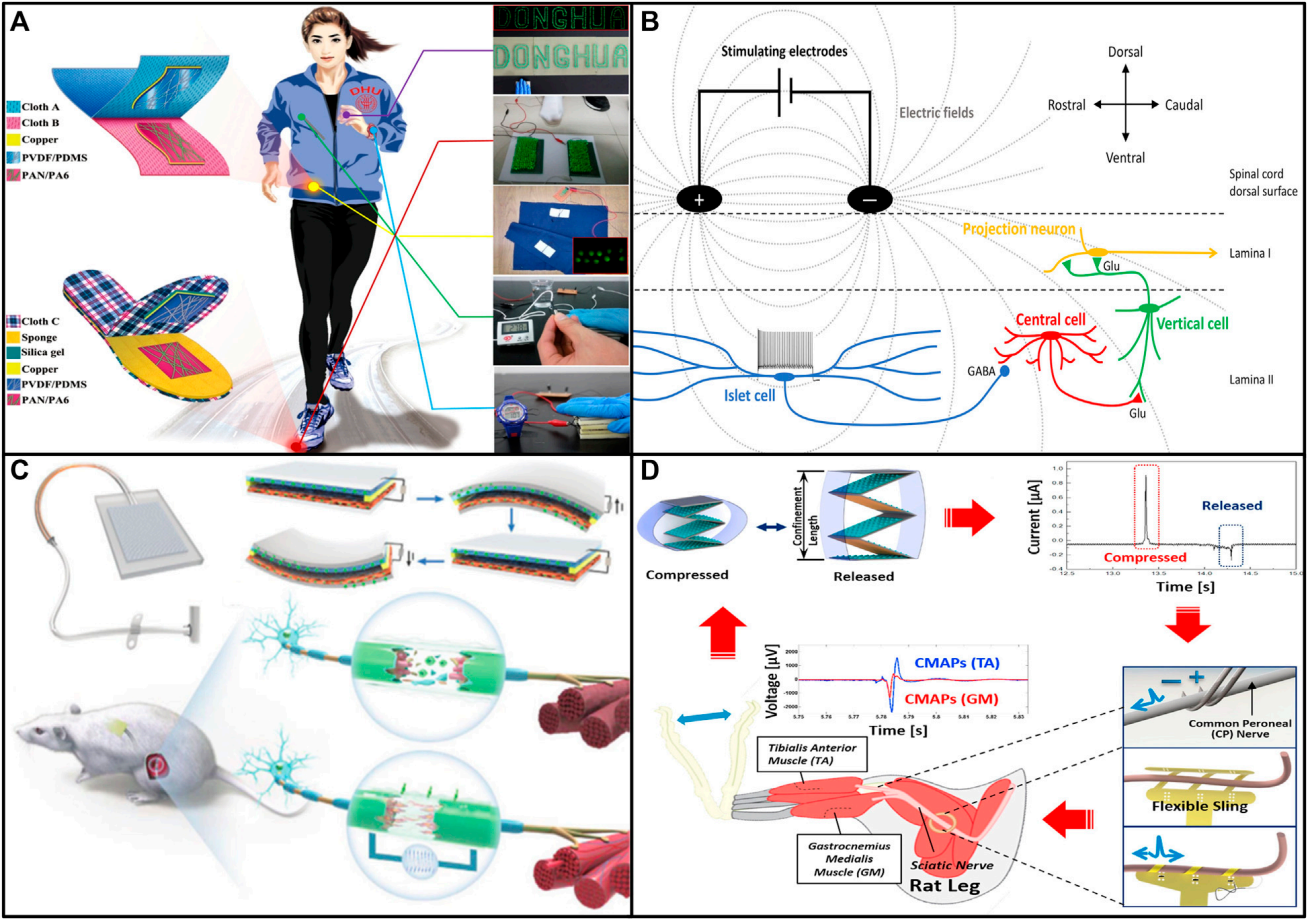
**(A)** Demonstration of the NM-TENG to harvest various biomechanical energy from human body. **(B)** Schematic illustrating proposed mechanism of action of spinal cord stimulation for pain. **(C)** Design and working principle of the ISR-NES system. **(D)** Schematic diagram of the conceptual system using flexible neural interfaces and TENGs (Lee et al., 2017; Li et al., 2017; Jensen and Brownstone, 2019; Zhou et al., 2022).

In order to power portable devices, scientists have conducted research on WTENG based on TENG. Li et al. optimized piezoelectric nanocomposites through high-throughput phase field simulation and machine learning, elucidated the fundamental mechanism of piezoelectric nanocomposites, and provided insights into the development of wearable electronics based on high-performance polymer/inorganic oxide composites a promising material design strategy (Li et al., 2022). In 2015, Kim et al. fabricated highly stretchable 2D fabrics by weaving fibers for fabric-structured TENG (FTENG), demonstrating stable high output voltage and current of 40 V and 210 μA (Kim et al., 2015). In 2019, Tang et al. designed a natural wearable cotton TENG (C-TENG) based on *in-situ* cotton on clothing, which can continuously power wearable electronic devices by efficiently converting biomechanical energy into electrical energy (Tang et al., 2020). Li et al. developed a lightweight, flexible and sustainable power source by fabricating a nanofibrous membrane constructed WTENG (NM-TENG), the model of NM-TENG is shown in Figure 2A The NM-TENG can convert human biomechanical energy into electrical energy for next-generation wearables and deliver a current and voltage output respectively up to 110 μA and 540 V (Li et al., 2017). Electrospinning is a general method for preparing micro/nanofibers. Nanofibrous membranes have the advantages of ultrathin diameter, good connectivity, easy scale-up, tunable wettability, fine flexibility, tunable porosity, and controllable composition and so on, which gives a great advantage when using it to make WTNG. In 2021, Su et al. constructed a polydopamine-modified nonwoven piezoelectric (PMNP) textile based on composite fibers, which exhibited excellent sensitivity and long term stability (Su et al., 2021b). Wei et al. propose a high-performance piezoresistive sensor based on MXene-Sponge for wearable biomonitoring and real-time tactile sensing (Wei et al., 2022).

### Development of TENGs in Medical Field and Neurostimulation

Nerve stimulation has become an important method for the treatment of SCI, its working principle is shown in Figure 2B. In 2009, DiMarco et al. discussed the use of phrenic nerve stimulation for spinal cord injury (Dimarco, 2009). Cecilia et al. successfully used transcutaneous electrical nerve stimulation for the treatment of neuropathic pain in spinal cord injury (Norrbrink, 2009). Moreno et al. discussed the use of electrical and magnetic nerve stimulation for the treatment of chronic pain in spinal cord injury using targeted therapy in 2014 (Moreno-Duarte et al., 2014). However, considering the current high cost of electrical spinal cord nerve stimulation and the complicated design of treatment methods and stimulators, it is necessary to find a new way to simplify the treatment plan and reduce the cost of electrical spinal cord nerve stimulation.

TENG has brought many changes to the medical field. There have been many articles summarizing the progress of TENG in the medical field (Kwon et al., 2004; Li et al., 2020; Parandeh et al., 2021). Air-driven TENG for self-powered real-time breathing monitoring proposed by Mao et al. (Wang et al., 2018) and a breathing human-computer interaction system based on it (Zhang et al., 2019). Wang et al. developed an integrated triboelectric self-powered respiratory sensor (TSRS) for simultaneous monitoring of human respiratory behavior and NH_3_ concentration in exhaled air (Wang et al., 2019; Yang et al., 2021). Liu et al. designed a breath-actuated triboelectric sensor for simultaneous breath biomechanical monitoring and exhaled acetone concentration analysis (Liu et al., 2022). Su et al. provide an overview of TENG-enabled self-powered respiration monitoring, focusing on the working principle, sensing materials, functional structures, and related applications in physical respiration motion detection and chemical respiration analysis (Su et al., 2021a). In 2021, Bhatia et al. proposed a wearable locomotion system for post-neural injury rehabilitation of the upper extremity based on TENG (Bhatia et al., 2021). Li et al. have carried out a series of studies on implantable TENG since 2014 and made great achievements, which proved the feasibility of using TENG to implant electrodes for electrical spinal cord nerve stimulation (Zheng et al., 2014; Tang et al., 2015b; Zheng et al., 2016a; Ma et al., 2016). In 2018, Long et al. reported a highly efficient electrical bandage for accelerated skin wound healing. On the bandage, WTENG alternating discrete electric fields by converting the mechanical displacement of skin motion into electrical energy (Long et al., 2018). Li et al. fabricated a series of biodegradable (Li et al.) implantable TENGs and successfully tuned their degradation process *in vivo* via near-infrared (NIR) photothermal manipulation. Degradation is triggered and takes effect within 12 h of NIR treatment. The output of BD implantable TENG can significantly accelerate the migration of fibroblasts in scratches, thereby enhancing the tissue repair process (Li et al., 2018). Jeong et al. introduced a wearable ionic TENG patch that delivers a uniform and symmetric electric field directly to the wound bed. In studies on normal human dermal fibroblasts, the electric field generated by ionic TENG was shown to accelerate cell migration, proliferation, and secretion of angiogenic growth factors *in vitro* and dermal fibroblasts from diabetic patients (Jeong et al., 2021). In 2021, Wan et al. established a flexible stretchable sandwich-structured triboelectric patch, composed of double-layer polydimethylsiloxane (PDMS) membrane with a hydrogel embedded for promoting biomechanical motion-driven wound healing. The bottom layer is compounded with Ag NWs to enhance the electric field between the electronic patch and the body. The hydrogel acts as an ionically conductive and stretchable electrode that can transport triboelectric charges to the Ag NWs. It has been demonstrated that periodic contact and separation between the cloth and PDMS layers caused by biomechanical motion significantly promotes wound healing (Wan et al., 2021).

Scientists have carried out a lot of research in the field of TENG for nerve stimulation, and the feasibility of the system has been successfully proved by experiments on animals. Zhou et al. developed an implantable self-regulating electrical nerve stimulation system based on the eccentric electrical nerve stimulation TENG they proposed previously, which can effectively promote synchronous regulation and regeneration of the sciatic nerve (Zhou et al., 2022). The working principle of this system is shown in Figure 2 (c). Yao et al. published a battery-free vagus nerve stimulation system that utilizes electrical impulses generated by a flexible TENG placed on the stomach in 2018 (Yao et al., 2018). Conta et al. proposed a TENG for therapeutic electrical stimulation (Conta et al., 2021). Li et al. reported several stacked Zig-Zag-shaped TENG units for neural stimulation through a tunable neural interface and demonstrated that they could successfully stimulate the sciatic and peroneal nerves in rats (Lee et al., 2017; Lee et al., 2019), and propose a conceptual schematic of the system used in the human body, which is shown in Figure 2 (d). Zheng et al. reported an implantable and biodegradable TENG for directed neural cell growth, demonstrating the feasibility of TENG for neuronal repair (Zheng et al., 2016b). In 2020, Guan et al. demonstrated a TENG-powered wireless neural stimulation electronic skin for characterizing synaptic plasticity (Guan et al., 2020). In 2022, Sun et al. proposed a closed-loop, self-powered and low-level vagal stimulation system based on a composite TENG, which effectively reduced the side effects caused by long-term vagal stimulation (Sun et al., 2022). In summary, the technology of TENG for neural stimulation is very mature, which has greatly promoted the development of neural stimulation.

### Advantages and Prospects of TENGs for Electrical Spinal Cord Nerve Stimulation

TENGs have high-voltage, low-current output characteristics, and their output currents are usually microamps (Wang, 2013; Zhang et al., 2019; Conta et al., 2021; Luo et al., 2021). A large number of wearable and implantable TENGs have also proved the safety and reliability of TENG on the human body. And the size of its output current can fully meet the current size required for nerve and spinal cord stimulation (Tang et al., 2015b; Li et al., 2017; Bhatia et al., 2021; Mathew et al., 2021). In addition, the design cost of the TENG is very low, and its use in electrical spinal cord nerve stimulation can solve the problem of expensive current electrical spinal cord nerve stimulation equipment, and can promote the popularization of electrical spinal cord nerve stimulation.

## Discussion

Electrical spinal cord nerve stimulation has been shown to relieve chronic pain to treat SCI (Shealy et al., 1967). But until now, electrical spinal cord nerve stimulation has been hindered by expensive equipment and complex treatment and stimulator designs (Jensen and Brownstone, 2019). But the rise of TENG has brought a new solution to this problem, using TENG to provide the electricity needed for electrical spinal cord nerve stimulation.

Nanofibrous membranes are highly flexible materials with the advantages of ultrathin diameters, good connectivity, easy scale-up, tunable wettability, fine flexibility, tunable porosity, and controllable composition, which make it very flexible and long-term to fabricate WTENG. In recent years, wearable and implantable TENGs have made great progress, which has also brought many changes to the medical field (Tang et al., 2015b; Li et al., 2017; Bhatia et al., 2021).

So far, there have been many studies on the use of TENG for nerve stimulation and SCI (Conta et al., 2021; Mathew et al., 2021), and through animal experiments, they have successfully repaired damaged nerves (Zheng et al., 2016b; Zhou et al., 2022), adjusted food intake to treat obesity (Yao et al., 2018), adjusted and controlled the anterior tibial bone (Lee et al., 2017), treatment of underactive bladder (Lee et al., 2019), represented the synaptic plasticity *in vivo* (Guan et al., 2020), treatment of atrial fibrillation (Sun et al., 2022). Combining TENG based on nanofiber membrane with nerve stimulation to relieve chronic pain disease and treat SCI will inevitably promote the development of nerve stimulation and give more patients the opportunity to recover. At the same time, we also need to consider the possible pitfalls of this type of treatment. The hard fibrotic foreign body-reactive tissue formed around such devices may prevent the TENG from working effectively. And electrical stimulation may cause localized heat shock, prolonged local inflammation, and electrical stimulation-related discomfort or pain (Conta et al., 2021).

## Data Availability

The original contributions presented in the study are included in the article/supplementary material, further inquiries can be directed to the corresponding author.
